# Community Connect: bridging the gap between scientific research and community engagement for TB elimination

**DOI:** 10.5588/pha.24.0005

**Published:** 2024-03-01

**Authors:** K. Ul Eman, M. S. Sivapuram, G. N. Kazi

**Affiliations:** ^1^ Dopasi Foundation, Pakistan, Islamabad, Pakistan;; ^2^ Communications and Engagement Consultant, Vijayawada, Andhra Pradesh, India;; ^3^ The Union, Paris, France.

**Keywords:** stigma, human rights, gender equity, person-centred care

The Union World Conference on Lung Health in November 2023 was a resounding success, marked by the remarkable attendance of over 4,500 delegates from around the globe. Held in the centre of Paris, the conference was significant as it was the first physical meeting for 4 years following the COVID-19 pandemic. The conference followed soon after the United Nations High-Level Meeting on TB held in September 2023,^1^ and had as its theme ‘Transforming Evidence into Practice’. The delegates, invigorated by the declarations made at the United Nations event, reflected a renewed and focused commitment to combating TB.

However, in spite of the recognised importance of communities in TB control,^2–6^ engagement throughout the care continuum remains limited. The Union has consistently prioritised community engagement via the Union's Community Advisory Panel (UCAP). This volunteer group comprises members from affected communities and civil society, and advises The Union’s board and management on how to address the needs of these communities. UCAP also coordinates Community Connect during the Union World Conference on Lung Health. Running in parallel to the scientific sessions, this dedicated segment amplifies the voices of those affected by TB and lung health issues, helping to share their experiences and highlight both the challenges and potential solutions. For this year’s conference, Community Connect blossomed into a dynamic platform with presentations from a diverse range of TB communities. The format fostered dialogue through panel sessions with engaging discussions and an exhibition area that transcended mere conversation to inspire concrete action. The sense of collective empowerment at Community Connect significantly elevated the lung health agenda, including the goal of eliminating TB by 2030. The featured sessions were organised under four distinct tracks,^7^ each addressing pivotal issues in community health and advocacy, including: 1) community rights, gender and stigma, focusing on innovative interventions promoting equitable access to TB and lung health services, while emphasising human rights, gender equity and removing stigma; 2) community engagement in pandemic preparedness and response, with valuable insights and best practices derived from combating COVID-19; 3) political contexts for community-led advocacy and accountability, emphasising translating strategic policy into concrete implementation, including sustainable financing required for TB elimination in the country context; and 4) driving equitable access to new tools and technologies, displaying how scientific advancements and digital innovations can coalesce with community empowerment in addressing poverty-related barriers and TB identification and elimination.

Various organisations across the globe were selected as track leads in liaison with UCAP to achieve their objectives. These leads created a large canvas to present a picture of the pivotal areas that can significantly contribute to TB elimination. These included: communities rights and gender (CRG) to enhance case notification; tobacco control (which is a gateway to several lung diseases, including TB); and non-communicable diseases such as cancer, diabetes, heart and chronic respiratory diseases (all major killers across the globe). In addition, there was coverage of mental health issues that can lead to morbidity, the need to leverage enhanced governmental funding at all levels, and social determinants to support and catalyse multi-sectoral accountability frameworks (MAF) at national and sub-national levels. To be effective, these efforts need to be undertaken with the active involvement of the communities affected, including via community-led monitoring. Community involvement and inter-sectoral action are vital cornerstones of the primary healthcare approach, but these are often ignored or considered too cumbersome. The infusion of new volunteers with fresh ideas about these crucial activities is likely to help drive these important elements and further catalyse TB control mechanisms. Building bridges such as Community Connect are crucial, especially given the time frame of only 6 years in which to meet the end TB targets. In line with the End TB Strategy's call for a people-centred response, community engagement and empowerment are more vital than ever. We need to invest in their future.

Community Connect is executing its mandate with remarkable dynamism and energy. If supported with adequate technical and material resources, it has great potential for the future. The passionate and informed presentations by representatives of TB-affected communities and civil society provided a beacon of hope. We need to recognise the importance of the role of the communities in translating the End-TB Strategy and Sustainable Development Goal 3 into reality and do everything possible to support their work.
